# A Mixed Methods Evaluation of a Whole Food Plant-Based Nutrition Program for Medical Students

**DOI:** 10.3390/ijerph23020194

**Published:** 2026-01-31

**Authors:** Tai Metzger, Deena Sukhon, Sophie Fisher, Zaheen Hossain, Virginia Uhley

**Affiliations:** Department of Foundational Medical Studies, Oakland University William Beaumont School of Medicine, Rochester, MI 48309, USAsdixon@oakland.edu (S.F.); zaheenhossain@oakland.edu (Z.H.);

**Keywords:** whole food plant-based diet, nutrition education, medical students, lifestyle medicine, dietary interventions

## Abstract

**Highlights:**

**Public health relevance—How does this work relate to a public health issue?**

- Diet-related chronic diseases, particularly cardiovascular disease and obesity, represent a leading and growing public health burden that is strongly influenced by modifiable dietary behaviors.
- Inadequate nutrition education among future physicians limits the healthcare system’s capacity to deliver effective, prevention-focused dietary counseling at the population level.

**Public health significance—Why is this work of significance to public health?**

- This study demonstrates that a short, whole food, plant-based (WFPB) nutrition intervention can significantly improve nutrition knowledge, attitudes, and readiness for lifestyle counseling among medical students.
- By strengthening physicians’ nutrition literacy and confidence early in training, this intervention addresses a critical upstream determinant of preventive care quality and chronic disease management.

**Public health implications—What are the key implications or messages for practitioners, policy makers, and/or researchers in public health?**

- Experiential, community-partnered nutrition programs may represent a scalable, low-cost strategy to enhance preventive health competencies within medical education and the broader healthcare workforce.
- Policymakers and educators should consider integrating practical, lifestyle-focused nutrition training into medical curricula to support long-term chronic disease prevention and health equity.

**Abstract:**

**Background/Objectives**: Whole food, plant-based (WFPB) diets have been associated with reduced cardiovascular risk and enhanced overall health. However, nutrition education in medical training remains limited. This study evaluated an experiential WFPB intervention known as the “Plant Plunge.” **Methods**: A total of 64 medical student participants attended weekly one-hour nutrition seminars on campus led by a local nonprofit, received complimentary WFPB lunches, and were encouraged to eat a WFPB diet for four weeks. Semi-structured interviews explored program perceptions. Pre- and post-intervention assessments measured nutrition knowledge, and a post-program survey assessed attitudes toward the intervention. **Results**: We analyzed a total of 14 interviews, 25 pre- and post-intervention knowledge assessments, and 49 post-intervention surveys. Qualitative analysis identified seven major themes: (1) improved physical health outcomes; (2) increased awareness of nutrition’s role in medicine; (3) concerns about feasibility and accessibility of WFPB diets; (4) personal empowerment and behavioral change; (5) educational value of seminars; (6) social engagement and peer support; and (7) relevance to future clinical practice. Mean scores on the knowledge assessment significantly improved from 73.3% to 87.0% (*p* = 0.045) following the Plant Plunge. Survey responses revealed that 65% of participants agreed that they increased knowledge of food ingredients, 54% indicated increased likelihood of selecting plant-based options, and 43% agreed that finding WFPB foods was easy, with 16% disagreeing. **Conclusions**: The Plant Plunge improved medical students’ nutrition knowledge, dietary attitudes, and perceived readiness for lifestyle counseling while offering an experiential model of nutrition education. Short, experiential nutrition programs may serve as scalable approaches to strengthen nutrition training and support chronic disease prevention.

## 1. Introduction

Chronic diseases such as cardiovascular disease, type 2 diabetes, hyperlipidemia, and obesity-related cancers represent the leading causes of morbidity and mortality in the United States and globally. A significant body of evidence points to poor dietary habits as one of the most modifiable and impactful contributors to this burden [1,2,3]. The standard American diet, characterized by high consumption of saturated fats, refined carbohydrates, added sugars, excessive sodium, and ultra-processed foods, has been consistently linked with the development and progression of these chronic conditions [1,2]. As healthcare systems continue to shift their focus toward preventive models, dietary interventions have emerged as a key strategy in mitigating risk and improving long-term health outcomes. Systematic reviews and meta-analyses have shown that even modest weight gain in adulthood is associated with significant increases in risk for multiple comorbidities [3].

The urgency of addressing dietary behavior is further underscored by the rapid and ongoing rise in global obesity rates. Since 1975, the worldwide prevalence of obesity has tripled, with current estimates indicating that nearly 60% of adults are classified as overweight or obese in some regions [4]. Evidence strongly supports that lifestyle modification—particularly through improved dietary patterns—offers a scalable, sustainable, and cost-effective approach to both primary and secondary prevention of chronic disease [1,2,3]. Among the dietary strategies gaining widespread attention for their health-promoting potential is the whole food, plant-based (WFPB) diet. This dietary pattern emphasizes the consumption of minimally processed plant-derived foods, including fruits, vegetables, legumes, whole grains, nuts, and seeds [2]. It differs from vegetarian or vegan diets in that it does not strictly exclude animal products but instead focuses on maximizing plant food intake while minimizing processed and high-fat animal-derived products [2,3]. Research has shown that rather than targeting individual nutrients or food items, broader adherence to healthy dietary patterns yields more robust and long-lasting health benefits, particularly in reducing the risk of cardiovascular disease [5].

The potential health benefits of WFPB diets are supported by an expanding body of clinical and epidemiological research [6,7,8,9,10]. Observational and interventional studies have found that individuals who adopt WFPB dietary patterns experience reductions in body weight and improvements in weight-related biomarkers [6,7]. In addition, long-term adherence to these dietary patterns has been associated with lower all-cause mortality and decreased cardiovascular disease incidence and mortality [9,10]. Moreover, WFPB diets have been shown to decrease the risk of developing type 2 diabetes and to support better glycemic control and metabolic regulation in patients already diagnosed with the condition [9,11]. These findings support the clinical utility of WFPB diets not only as a preventive strategy but also as an adjunctive treatment in managing chronic illnesses.

Despite the wealth of evidence highlighting the importance of nutrition in health and disease, medical education has not kept pace with this paradigm shift. Major medical and professional organizations, including the American Society for Clinical Nutrition and the American Medical Student Association, have repeatedly emphasized the necessity of integrating comprehensive nutrition training into the medical school curriculum [12]. However, surveys and curriculum reviews indicate that current nutrition education remains insufficient and inconsistently applied across medical schools [13,14]. For example, many institutions do not meet the National Academy of Sciences recommended minimum of 25 h of nutrition instruction during medical school [15,16].

A closer look at the structure of nutrition education reveals further challenges. In a multi-institutional review of 30 medical schools, the majority of nutrition-related content was delivered through didactic lectures (33%) or stand-alone sessions (57%), with only 10% offered as electives [17]. The Nutrition Education in Medical Schools project identified notable gaps between what is taught and what is needed in clinical practice, advocating for a multidisciplinary approach to teach basic, applied, and clinical nutrition [18]. The lack of robust, experiential learning opportunities in medical school contributes to a gap in knowledge and confidence among physicians, hindering their ability to deliver nutritional care effectively [19,20,21]. The importance of addressing this gap is compounded because medical students themselves often struggle with maintaining healthy dietary habits. One study found that only 20% of medical students met the World Health Organization’s recommendation of consumption of fruits and vegetables [22]. Moreover, their dietary patterns tended to be high in sodium and animal fats [22]. The COVID-19 pandemic further exacerbated these trends, leading to increased consumption of fast food and energy drinks [23].

Encouragingly, recent interventions have shown that even brief, structured nutrition education programs can have a meaningful impact. In one experimental study, a four-week nutrition curriculum significantly improved students’ nutrition knowledge, confidence in counseling, dietary diversity, and consumption of fruits and vegetables [24]. By integrating nutrition education into both the formal curriculum and co-curricular activities, medical schools can empower students to adopt healthier lifestyles and enhance their capacity to deliver evidence-based dietary counseling in clinical practice.

In response to these needs, our study sought to evaluate the impact of a four-week WFPB dietary intervention and nutrition seminar series, known as the Plant Plunge, on medical students’ dietary knowledge and perceptions of nutrition education. Organized in partnership with Chickpea and Bean, a nonprofit dedicated to promoting plant-based nutrition, the Plant Plunge combined self-directed dietary changes, structured seminars, and biometric health assessments of participants blood pressure, weight, cholesterol, and glucose. Our aim was to explore participants’ perspectives on the value of the Plant Plunge nutrition seminars and their broader views on nutrition education within their medical training. This study contributes to the growing body of literature advocating for integrated, practical nutrition education in medical school and offers insights into the potential health benefits of plant-based eating among future healthcare providers.

## 2. Materials and Methods

### 2.1. Program Overview

**Figure 1:**
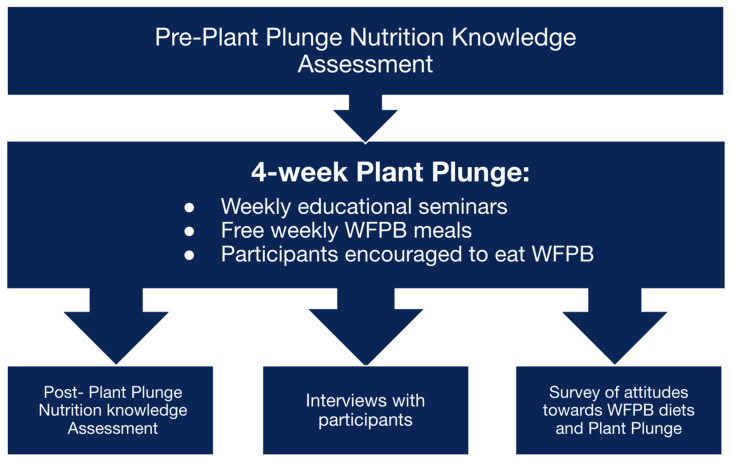
Study Design. We performed a mixed methods evaluation of a four-week WFPB diet intervention.

“Plant Plunge” was a prospective, observational intervention conducted at Oakland University William Beaumont School of Medicine (OUWB), an allopathic (M.D.) medical school, in collaboration with Chickpea and Bean, a minority-led nonprofit based in Detroit (chickpeaandbean.com accessed on 18 December 2025). The organization is dedicated to promoting the benefits of WFPB nutrition through community engagement and education. Chickpea and Bean designed the Plant Plunge as a four-week intervention to increase awareness and adoption of plant-based dietary patterns while enhancing nutrition education for medical students. Methods are summarized in Figure 1.

The total population of medical students available for recruitment was 250, and all were invited to voluntarily participate in the Plant Plunge program, which was delivered in-person and free of cost. The program was advertised via GroupMe messages sent to the GroupMe chats of all OUWB classes. We did not have an a priori sample size goal. The program included three core components: (1) a personal challenge to eat a WFPB diet; (2) weekly nutrition seminars; and (3) biometric health screenings before and after the intervention, including weight, blood pressure, glucose, and cholesterol concentration. Glucose and cholesterol concentrations were measured using point-of-care machines from finger-prick collection rather than blood draw and were immediately available to participants. Biometric data was collected so that participants could see their own improvements, rather than for analysis in this paper. However, information on the biometric data was indirectly analyzed through references to these measurements during interviews.

Nutrition seminars were held weekly for four weeks, each one hour in duration and paired with a complimentary WFPB meal. The sessions took place in a lecture-style format and were facilitated by Chickpea and Bean educators and guest clinicians. A four-week intervention was selected in order to give participants sufficient time to experience eating a WFPB diet without having to make a long-term commitment greater than one month. This length also gave sufficient time to cover the content over the course of four, weekly sessions and fit with the budget and schedule of Chickpea and Bean. The content of the curriculum was developed by Chickpea and Bean based on the educational content that they provide in the community and consisted of PowerPoint presentations (version 16.105.2). Sessions were hosted in a classroom on campus. The vegan lunches were purchased from local restaurants by Chickpea and Bean and included Mexican sopes, Mediterranean rice bowls with falafel and hummus, pizza with vegan cheese and pepperoni, and vegetable wraps. Recipes for these meals were not provided.

The seminar content was structured around progressive educational goals. Week 1 introduced the program and included patient narratives from individuals who had reversed chronic disease through a WFPB lifestyle. Week 2 covered nutrition label literacy, grocery shopping strategies, and common food misconceptions. Week 3 addressed social and environmental barriers to plant-based eating, including cost, dining out, and peer influence, and included example recipe. Week 4 featured a board-certified internist trained in integrative and lifestyle medicine, who presented on the clinical applications of WFPB nutrition and led an open Q&A discussion.

Key outcomes were nutrition knowledge measured using pre- and post-Plant Plunge knowledge assessments, attitudes toward plant-based eating and the Plant Plunge using semi-structured interviews, and intention to change diets using a post-intervention survey. Demographic information was not collected. Participation in the interviews, pre-and post- assessments, and survey was not required and was open to any attendees who chose to participate. Participants were encouraged but not required to attend all four seminars.

### 2.2. Participant Recruitment

Participants were recruited through student listservs, class announcements, and student-led interest groups. All medical students were eligible to participate regardless of year or prior nutrition experience. Participation was voluntary and not incentivized. Students who enrolled provided verbal informed consent. De-identified data were stored in a secure Google Drive folder accessible only to the research team.

### 2.3. Interview-Based Data Collection

To further explore student perceptions of the program, participants were invited to participate in brief, semi-structured interviews (Appendix A). Because the Plant Plunge represented a relatively novel educational and dietary intervention, qualitative interviews allowed us to explore unanticipated themes and better understand the factors influencing its impact. Semi-structured interviews were used to capture nuanced, open-ended responses that could not be fully assessed through quantitative measures, providing insight into participants’ experiences and perceptions. The interview guide was developed by the study authors in collaboration with expert faculty and the Plant Plunge coordinators. We asked participants about their experiences with the Plant Plunge, attitudes toward the 4-week program, the educational content of the seminars, changes in their pre- and post- intervention measures (weight, blood pressure, glucose, and cholesterol concentrations), perceptions of medical school nutrition education, lifestyle medicine, personal attitudes toward plant-based eating, and dietary habits. Interviews were conducted by trained student researchers, recorded with consent, and transcribed verbatim. Two independent reviewers used thematic analysis to code transcripts. Interviews were analyzed for themes in an iterative process until percent agreement between reviewers reached 80%. Coding continued until thematic saturation was reached, defined as the point at which no new concepts or themes emerged.

### 2.4. Pre- and Post-Program Knowledge Assessment

To assess the educational impact of the seminar series, participants completed a 10-question knowledge assessment before and after the four-week intervention. The assessment was created by the authors of the study and reviewed by expert faculty and the Plant Plunge hosts to ensure validity. The questions evaluated students’ understanding of WFPB nutrition, interpretation of food labels, and knowledge of dietary risk factors for chronic disease. The same instrument was administered pre- and post-intervention using anonymous codes. Data were analyzed using a two-tailed *t*-test to determine whether the observed differences in mean scores were statistically significant. The knowledge assessment can be found in Appendix B.

The pre- and post-intervention knowledge assessment was a purpose-built instrument developed to evaluate learning objectives specific to the Plant Plunge curriculum, including whole food, plant-based (WFPB) nutrition principles, food label interpretation, and dietary risk factors for chronic disease. Because no existing validated questionnaire fully captured these targeted domains in medical trainees, the assessment was developed using a content-validity–driven approach [25,26]. Initial items were drafted based on seminar objectives and the nutrition education literature, then reviewed and refined through expert evaluation by faculty with experience in nutrition education and lifestyle medicine, as well as program educators, to ensure relevance, clarity, and alignment with evidence-based concepts. Face validity was supported by the direct correspondence between assessment items and instructional content. Preliminary construct validity is suggested by the improvement in scores following the intervention, indicating sensitivity to educational change consistent with established methods for evaluating short-term educational interventions [25,26].

### 2.5. Post-Intervention Survey on Attitudes and Perceptions

At the end of the program, all participants were asked to complete an anonymous post-intervention survey. The short survey assessed student attitudes toward the Plant Plunge program and plant-based eating. Responses were collected using a 5-point Likert scale (1 = strongly disagree, 5 = strongly agree) and analyzed descriptively.

The post-intervention survey assessing attitudes and perceptions toward the Plant Plunge and plant-based eating was developed using expert-informed content validation [25,26]. Survey items were designed to capture key attitudinal constructs addressed during the program, including perceived nutrition knowledge, intention to choose plant-based foods, and feasibility of adopting WFPB dietary patterns. Draft items were reviewed by the study team and faculty experts to ensure conceptual clarity, appropriateness for medical students, and alignment with program goals consistent with survey best practices in medical education developed by International Association for Health Professions Education (AMEE) [27]. Face validity was supported by the use of clear, behaviorally anchored Likert-scale statements directly reflecting participant experiences during the intervention. Construct validity was further supported through triangulation with qualitative interview findings, which demonstrated thematic consistency between survey responses and in-depth participant narratives. Given the exploratory nature of this pilot study, the survey was intended to assess perceived impact rather than serve as a fully standardized psychometric instrument with formal reliability testing and factor analysis in which a larger sample is needed.

## 3. Results

### 3.1. Interviews

A total of 64 students participated in the plant plunge (26% of the total student body), and 30 had biometric data obtained. A total of 14 interviews were conducted. The interviews revealed seven recurring themes about the participants’ personal and professional growth (Table 1). Nine students emphasized how the program deepened their understanding of nutrition and introduced them to the health benefits of a WFPB diet. Participants described learning new and practical information about food labels, plant-based substitutions, and the role of diet in managing and preventing chronic conditions. For some, the experience corrected misconceptions about plant-based nutrition and empowered them to take greater ownership of their health. This was particularly impactful for students who reported improvements in objective health markers such as weight loss, cholesterol reduction, or improved digestion. According to six interviewees, these outcomes not only reinforced the value of dietary change but also motivated ongoing behavioral adjustments beyond the four-week program.

Students frequently highlighted the gap between their nutrition education during medical school and the real-world applicability of what they learned during the Plant Plunge. Six participants reflected that they had not previously been exposed to this type of information in their medical curriculum, despite its clear relevance to patient care. The program was viewed as a much-needed supplement to traditional medical training, with students calling attention to the lack of structured nutrition instruction and the missed opportunity this presents for future physicians. Seven participants expressed a newfound commitment to integrating dietary counseling into their future clinical practice, acknowledging that current medical education often prioritizes pharmacologic treatment over preventive, lifestyle-based strategies. The seminar series provided by Chickpea and Bean was praised for being understandable, evidence-based, and immediately applicable—qualities that students said were rarely found in formal coursework.

Despite the overall positive feedback, participants acknowledged several challenges in adopting and maintaining a WFPB lifestyle. While seven participants found the vegan meals surprisingly enjoyable, four noted initial concerns about the effort and accessibility required to make dietary changes, particularly around cost, convenience, and finding suitable substitutes for familiar foods. Nevertheless, five students reported that these barriers were easier to overcome than expected, especially with the help of peer support and increased exposure to plant-based options. 

### 3.2. Nutrition Knowledge Assessment

There were a total of 25 completed pre- and post-assessments of nutritional knowledge. We found a statistically significant increase in scores on the assessment from 73.3% to 87.0% following the Plant Plunge (*p* = 0.045).

### 3.3. Survey

**Figure 2:**
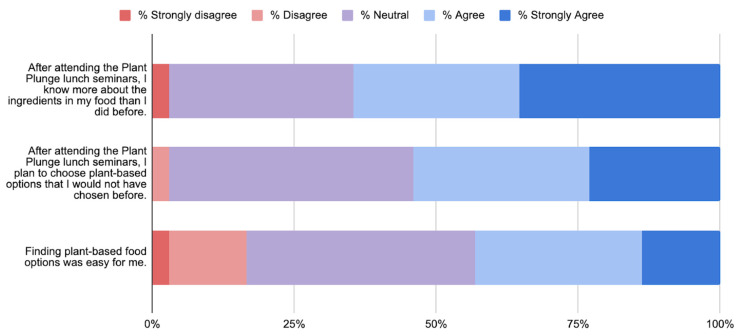
Survey responses.

There were a total of 49 survey participants (summarized in Figure 2). 65% agreed or strongly agreed with the statement “After attending the Plant Plunge lunch seminars, I know more about the ingredients in my food than I did before.” 54% agreed or strongly agreed with the statement “After attending the Plant Plunge lunch seminars, I plan to choose plant-based options that I would not have chosen before.” Only 1 student disagreed with the statement, and the rest were neutral. 43% agreed with the statement “Finding plant-based food options was easy for me.” 16% disagreed or strongly disagreed, with the rest being neutral.

## 4. Discussion

This study evaluated the impact of a four-week whole food, plant-based (WFPB) intervention—the Plant Plunge—on medical students’ nutrition knowledge, perceptions of medical education, and dietary attitudes. While previous research has shown the beneficial health effects of WFPB, our study provides new findings regarding the impact of an experiential WFPB educational program for medical students. Interviews highlighted a range of physical improvements, including better digestion, weight loss, reduced low-density lipoprotein (LDL) cholesterol, and enhanced energy levels. Students expressed surprise at how accessible and enjoyable WFPB eating could be, often commenting on the variety and taste of the plant-based meals. Many also reported behavioral shifts, such as continued interest in WFPB eating after the program’s conclusion. Post-intervention assessments revealed a statistically significant increase in nutrition knowledge. Survey results showed increased awareness of food ingredients, greater likelihood of choosing plant-based options, and mixed attitudes towards feasibility of implementing a plant-based diet.

Beyond personal health outcomes, the intervention also appeared to fill a notable gap in formal medical education. Interviewees consistently emphasized how the Plant Plunge offered practical, evidence-based nutrition education that was largely absent from their existing curriculum. Students valued the seminar content for its clarity and clinical relevance, and several stated that it improved their confidence in counseling patients on lifestyle changes. These findings align with earlier research by Coppoolse et al., who found that short-term nutrition interventions improved both knowledge and intent to counsel among medical students in the Netherlands [14]. Similarly, Bassin et al. described the widespread inadequacy of nutrition training in U.S. medical schools, noting that most programs fail to meet recommended minimums for nutrition instruction [17].

Our findings also support existing literature on the clinical efficacy of WFPB interventions. Bansal et al. demonstrated that group-based WFPB diet programs can significantly improve health outcomes in underserved patient populations, reinforcing the importance of dietary counseling as a public health strategy to combat chronic diseases [28]. This aligns with participants’ recognition of diet as a modifiable risk factor for chronic disease, and their expressed desire to integrate nutrition into their future clinical practice. Additional studies further validate these findings. For instance, Crowley et al. emphasized the role of practical, skill-based nutrition education in bridging the gap between knowledge and behavior [11], while a recent intervention by Amoore et al. showed that even short curricula can boost medical students’ dietary habits and counseling self-efficacy [24]. Moreover, medical students’ firsthand experiences with dietary change may serve as a catalyst for enhancing empathy and credibility in future patient counseling. By personally navigating facilitators and barriers to adopting a WFPB diet, such as feasibility concerns, social influences, food access, and perceived health changes, students gain practical insight into the lived challenges patients face when modifying dietary habits. This experiential understanding may translate into more nuanced, patient-centered counseling strategies, greater appreciation for structural determinants of nutrition, and a stronger commitment to advocating for accessible, culturally adaptable dietary interventions in clinical settings.

Despite these promising results, our study has several limitations. First, it was conducted at a single institution with a relatively small, self-selecting sample of medical students, which may limit generalizability. Participation was voluntary and may have attracted students with preexisting interest in nutrition or preventive medicine. While it is difficult to generalize the results to the general population, we believe it is useful to show the impact of this program on students who are already open to learning more about nutrition. There was also no control group and demographic data was not collected, making it difficult to ensure that effects were due to solely to the Plant Plunge. Additionally, biometric outcomes, such as weight loss, were self-reported, and long-term adherence to dietary changes was not assessed. These factors introduce potential biases and limit conclusions about sustained impact.

Future research should aim to replicate these findings in larger, more diverse medical student populations and evaluate long-term effects on dietary behavior and clinical application. Randomized controlled trials comparing different formats and durations of nutrition education could help identify optimal approaches. Additionally, integrating standardized nutrition competencies into the core curriculum—beyond elective or co-curricular offerings—may yield broader and more consistent improvements in preparedness to deliver nutrition care.

## 5. Conclusions

Ultimately, the Plant Plunge served as an effective experiential education model, equipping future physicians with practical skills and reinforcing the clinical importance of nutrition. Participants not only reported improved health and increased knowledge but also developed greater confidence in their ability to apply nutrition in patient care. As the burden of diet-related chronic disease continues to grow, these findings underscore an urgent need to incorporate lifestyle-focused, WFPB-centered education into mainstream medical training. Doing so may help cultivate a generation of physicians better prepared to address the root causes of chronic illness.

## Figures and Tables

**Table 1:** Common themes and example quotations from interviews.

Theme	Definition	Example Quotes
**Improved Physical Health Outcomes**	Participants experienced tangible health improvements such as weight loss, improved digestion, and better lab values.	“I was able to drop my LDL in half, so I’m really, I was really excited about that. And just like overall, I just felt like better in general.” “I did find in this process that I’m not worried about my cholesterol. I’m not worried about my triglycerides. My blood pressure is great.” “I noticed, especially when I got my lab values back, that my cholesterol and a lot of those bad values that were kind of high before went down in just four weeks, which I thought was really interesting because being in my 20s, I didn’t really think about my blood work that much and kind of assumed that I was living a pretty healthy lifestyle.”
**Increased Awareness of Nutrition’s Role in Medicine**	Students gained insight into how nutrition directly connects to patient care and the practice of medicine.	“It doesn’t matter how many hours we spend learning these drugs, like statins, to improve our blood and our cardiovascular system if we cannot take care of the diet.” “I think that nutrition education is really important in medical school. I think the potential benefits of a plant-focused, or at least a minimal meat-consuming diet, are really significant.”
**Feasibility and Accessibility of Plant-Based Diets**	Participants found plant-based eating to be easier and more sustainable than expected, often noting available alternatives.	“It’s a lot easier than I thought it would be to like make those changes and still enjoy food.” “It was my first time committing to eating fully vegan for a few weeks, so I was a bit intimidated by it at first, but as soon as I started, I realized how feasible it was. And we learned from Chickpea and Bean that it can still be affordable and you can still eat foods that taste good and that fuel your body.” “Nowadays there’s so many alternative options for you know if you’re trying to eat vegetarian or vegan there’s a lot of options these days. So I didn’t find it as challenging as I thought it was going to be.”
**Personal Empowerment and Behavioral Change**	Students described feeling motivated to continue plant-based eating and apply these habits long-term.	“To see that change in four weeks definitely is going to affect how I eat on the regular and kind of trying to implement plant-based food in my life.” “I hope that, you know, we continue this plant based, plant based initiative so that, you know, we could continue lowering our LDL levels, decreasing our, decreasing our blood pressure and making sure, you know, making sure that our blood glucose levels are, are within range so that we don’t develop type two diabetes.”
**Educational Value of the Seminars**	Seminars were appreciated for being informative, accessible, and relevant to both personal and professional growth.	“I learned how like it’s like shifting more towards a plant-based diet is important, especially if you have a family history of like, you know, type 2 diabetes, hypertension, hyperlipidemia.” “The info sessions were really enjoyable, and I learned a lot from the sessions.” “I also learned a bit more about healthy fats versus unhealthy fats and how you can change, you know, the composition of the fats in your diet. It does make a difference.”
**Positive Social Engagement and Peer Support**	Participants valued the communal nature of the program, bonding with peers and feeling supported by classmates.	“Connecting with other classmates and seeing how they’ve been progressing throughout the four weeks and seeing all their improvement has really made me happy and has really inspired me.” “The info sessions were really enjoyable with my classmates, and I learned a lot from the sessions, even though it wasn’t too intense.”
**Relevance to Future Clinical Practice**	Students recognized the long-term applicability of plant-based knowledge to their role as future physicians.	“I can definitely use a lot of information with my patients going forward and trying to help them have better lifestyles and better diets.” “In terms of nutrition education, I do think it’s crucial, especially in medical school, since I will be joining the workforce and helping future patients navigate their diets.” “It was really cool for us to learn about how eating a plant-based diet can really lower your risk of chronic disease or if you have chronic disease it can really help with your symptoms. So I think moving forward just having this knowledge is really helpful for us so that we can help our future patients.”

## Data Availability

The data presented in this study are available on request from the corresponding author due to privacy of participants.

## Abbreviations

The following abbreviations are used in this manuscript:

WFPB	Whole food, plant-based
LDL	Low-density lipoprotein

## Appendix A

Semi-structured interview format:

1. “Did you enjoy the plant plunge?”
2. “What did you enjoy about it?”
3. “What did you learn about heart-healthy diets from the plant plunge?”
4. “How were your thoughts on whole food plant-based diet and other heart-healthy diets changed after the Plant Plunge?”
5. “What do you think about the importance of nutrition education in medical school?”
6. “What changes would you make to nutrition education in medical school curriculum?”
7. “How will you implement nutrition into your future practice?”

## Appendix B

1. Which of the following diets has been shown to significantly lower LDL cholesterol and reduce the risk of cardiovascular disease?

☐ High-protein ketogenic diet

☐ Low-fat plant-based diet

☐ Paleo diet

☐ Standard Western diet

2. What percentage of cardiovascular disease cases are estimated to be preventable through lifestyle changes?

☐ 10–20%

☐ 30–40%

☐ 50–70%

☐ 80–90%

3. Which of the following is NOT a benefit of a low-fat plant-based diet for heart health?

☐ Lowering blood pressure

☐ Improving arterial function

☐ Increasing saturated fat levels

☐ Reducing inflammation

4. Which dietary component has been most strongly linked to increased CVD risk?

☐ Monounsaturated fats

☐ Saturated fats

☐ Omega-3 fatty acids

☐ Complex carbohydrates

5. How does excess sodium intake increase cardiovascular risk?

☐ By increasing blood viscosity

☐ By raising blood pressure

☐ By decreasing HDL cholesterol

☐ By lowering cardiac output

6. What effect does a low-fat plant-based diet have on endothelial function?

☐ It has no effect

☐ It impairs endothelial function

☐ It improves endothelial function

☐ It reverses endothelial damage

7. Which of the following has the greatest impact on reducing cardiovascular disease risk?

☐ Pharmacological interventions

☐ Genetic testing

☐ Lifestyle modifications

☐ Regular health check-ups

8. Which factor contributes most to the development of metabolic syndrome, a major risk factor for cardiovascular disease?

☐ Excess sugar intake

☐ Smoking

☐ Lack of physical activity

☐ Both a and c

9. Which of the following nutrients indicates that a food contains animal products?

☐ Omega fatty acids

☐ Vitamin B1

☐ Cholesterol

☐ Vitamin D

10. Which of the following types of food is most useful for feeling full while consuming fewer calories?

☐ High volume, low calories foods such as nuts and beans

☐ High volume, low calories foods such as vegetables

☐ Low volume, high calorie foods such as nuts and beans

☐ Low volume, high calorie foods such as vegetables
